# Effect of different orientations of screw fixation for radial head fractures: a biomechanical comparison

**DOI:** 10.1186/s13018-017-0641-9

**Published:** 2017-10-02

**Authors:** Xuchao Shi, Tianlong Pan, Dengying Wu, Ningyu Cai, Rong Chen, Bin Li, Rui Zhang, Chengwei Zhou, Jun Pan

**Affiliations:** Department of Orthopaedics Surgery, The Second Affiliated Hospital and Yuying Children’s Hospital of Wenzhou Medical University, No.109, Xue Yuan West Road, Wenzhou, Zhejiang Province 325027 China

**Keywords:** Radial head fractures, Screw, Biomechanical comparison, Different orientation

## Abstract

**Background:**

Screw fixation is a common method used for the treatment of Mason type II radial head fractures. The purpose of our study was to evaluate the mechanical properties of three different screw orientations used for fixation of Mason type II radial head fractures.

**Methods:**

We sawed 24 medium-frequency fourth-generation Synbone radial bones to simulate unstable radial head fractures, which we then fixed with three different screw orientations. Implants were tested under axial load by the tension-torsion composite test system. If the implant-radial constructs did not fail after the axial load test, an axial failure load was added to the remaining constructs.

**Results:**

The stiffness of the divergent group was the highest of the three orientations, and this group had statistically significant difference from the other two groups (*p* < 0.05). However, there was no statistically significant difference between the convergence group and the parallel group (*p* > 0.05). When the displacement reached 2 mm, the load of the divergent screw was still larger than the other two groups (*p* < 0.05).

**Conclusions:**

The divergent screw orientation was the most stable and had the greatest control of Mason type II fractures of these three groups. Therefore, it can be better applied in clinical settings.

## Background

Mason type was proposed for the first time in 1954 as a definition of radial head fractures [1]. Mason type II fractures were 2-part fractures of the radial head with displacement and then were modified by Broberg and Morrey [2] as having more than 2 mm of displacement and involving at least 30% of the radial head. The optimal treatment for Mason type II fractures of the radial head is still controversial [3, 4]. Radial head fractures are infrequently seen in adults, with a reported incidence of approximately 55.4 per 100,000 people [5]. The mechanism of injury in radial head fractures is usually caused by the arm reaching in a fall, and in a few cases it is caused by direct violence [1, 6]. Fortunately, type II radial head fractures can be managed with conservative treatment when the displacement is less than 2 mm [3]. Although the management of displaced radial head fractures in adults remains unsatisfactory due to problems with loss of reduction, and non-union [6–12], there has recently been a great interest in preserving the radial head using open reduction with internal fixation. Since the important role of the radial head in elbow stability has been recognized [12], special implants for the maintenance of radial head fractures have been developed to improve fixation stability.

In clinical practice, two headless compression screws placed parallel to each other are used [13]. However, there is no biomechanical data regarding this common fixation method if the screws occur to be placed in a non-parallel orientation. The purpose of this study was to quantify and compare the stiffness of three different screw configurations used to stabilize a simulated Mason type II fracture.

## Methods

**Fig. 1:**
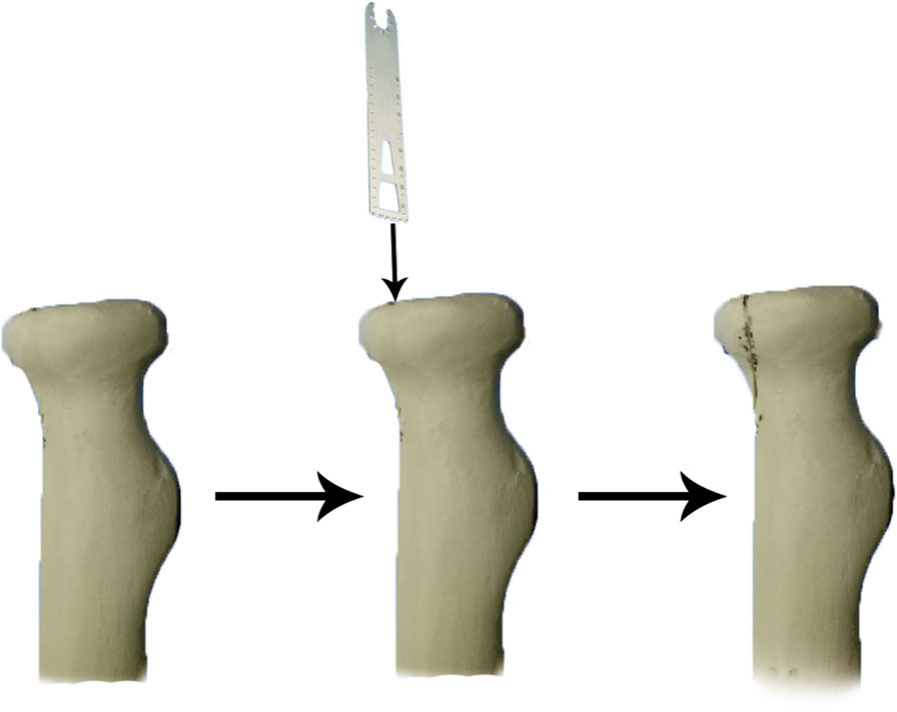
The production process of Mason II radial head fractures

**Fig. 2:**
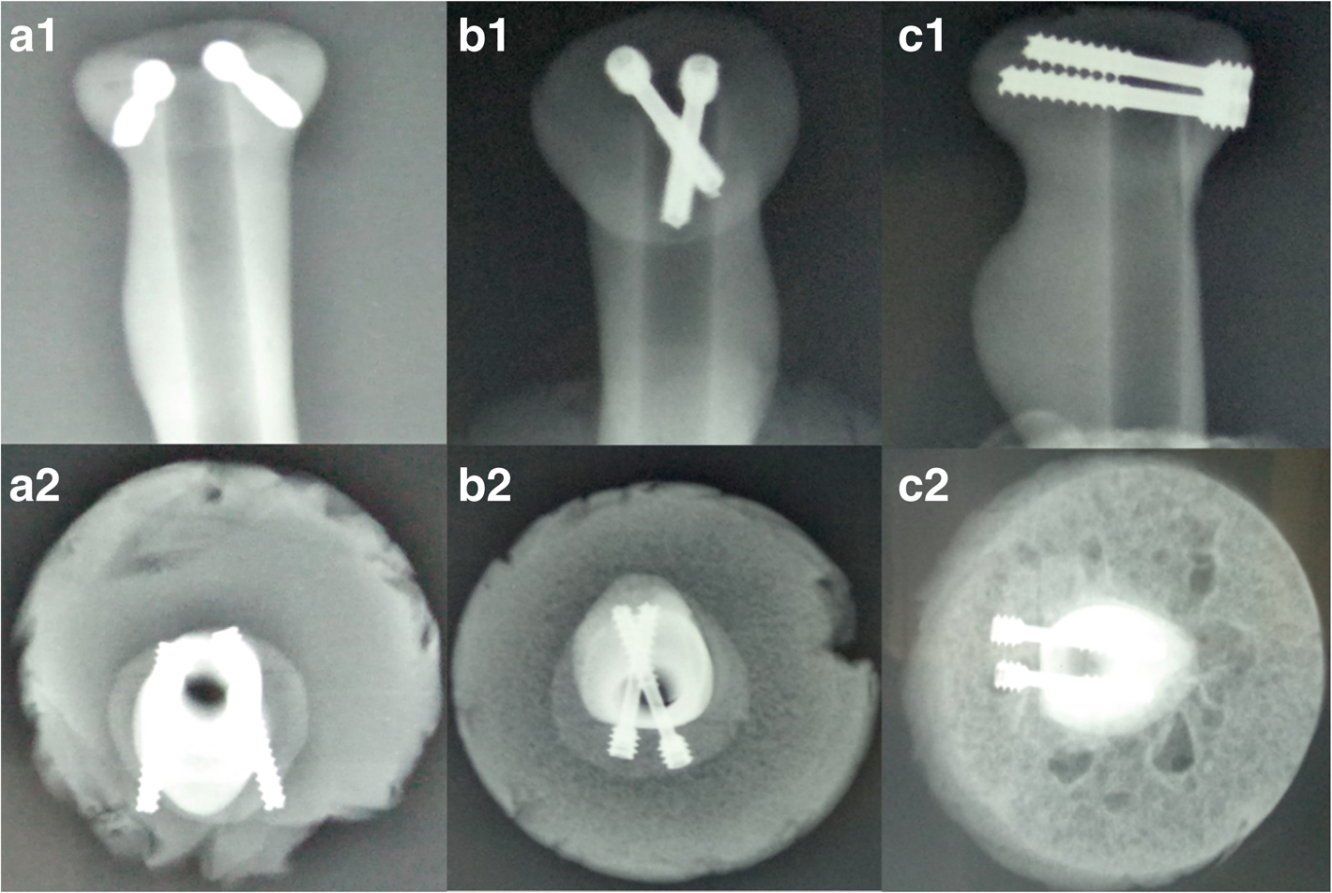
Radiographs of convergent group, parallel group, and divergent group

Twenty-four Synbone radial bones (Malans Synbone Company, Switzerland) of the same size and density were used. The radius was cut mid-shaft, leaving an approximately 10-cm-long proximal segment. A Mason type II fracture was produced (Fig. 1). The fracture was created with an oscillating saw parallel to the longitudinal axis of the specimen. With this fragment size, the fracture ended at the radial neck without any bony support. The fragment included the safe zone that is the part of the radial head that does not articulate with the proximal radioulnar joint. Reduction was performed and maintained with a reposition clamp, and the screws were implanted. We used two screws (Wright, Beijing, China) to fix the fracture model. The three different orientations were as follows: (1) convergent group: two screws were inserted 30 ° convergent to each other in the transverse plane; (2) parallel group: two screws were inserted parallel to each other and perpendicular to the fracture line; (3) divergent group: two screws were inserted 30 ° divergent to each other in the transverse plane. Figure 2 shows X-rays of the reconstructed radial heads with the three screw fixations described above. The screws were inserted 5 mm proximal to the top of the radial head. The screws were all spaced 5 mm apart at the medial cortex regardless of orientation.

One fellowship-trained orthopedic surgeon performed the fixation on all 24 specimens to minimize the variability of fixation parameters. The Synbone surfaces were smooth, and the initial reduction was only accepted if it was anatomical. The clamps and drills used were the same in all three experimental groups. The transversely cut end of the radial shaft was potted in a metal tube using polymethylmethacrylate. The average length of the proximal radius exposed outside of the potting material was 6.79 cm, and the average diameter of the radial head was 2.28 cm. As in clinical use, the length of the screws did not exceed the contralateral cortex.

**Fig. 3:**
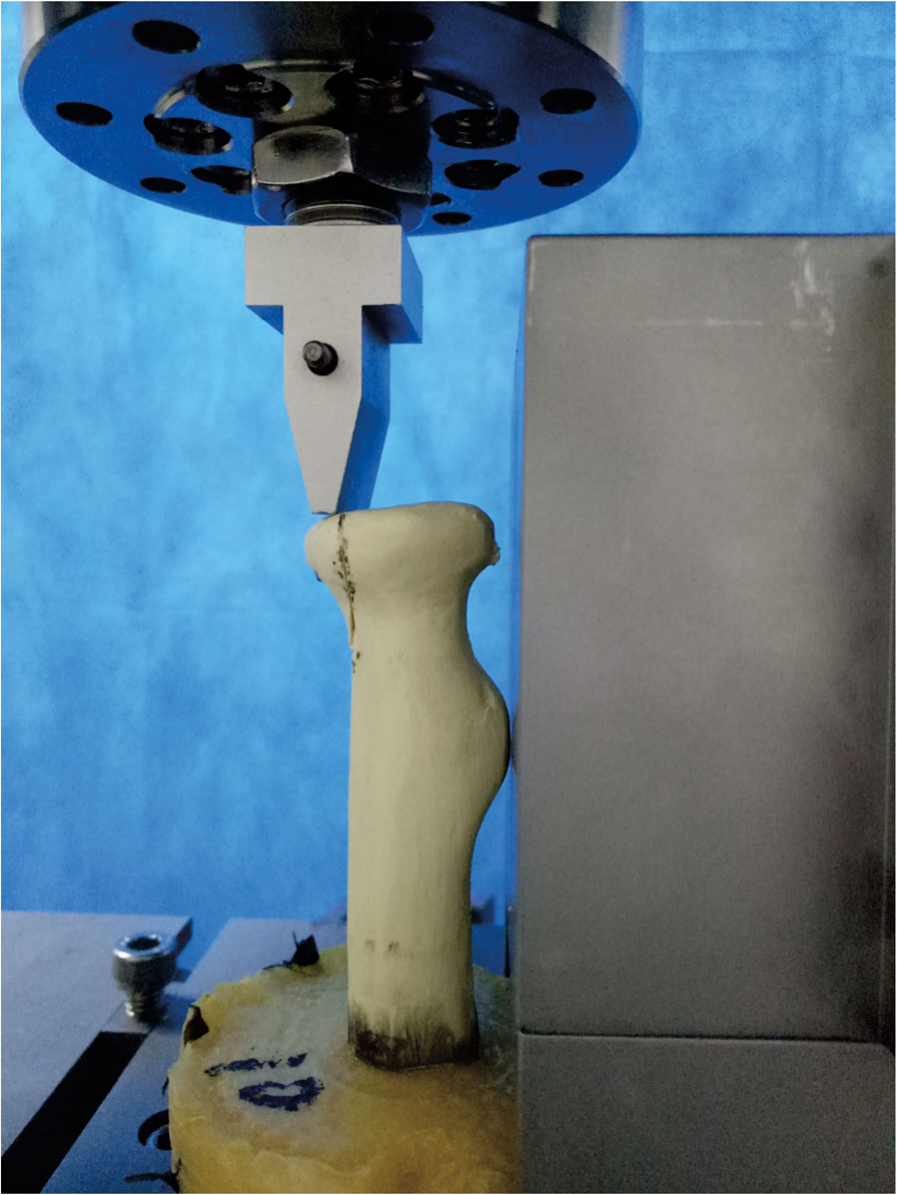
The radial head model was placed in the instrument for axial loading

The axial load was applied to the radial head fragment through a metal block (Fig. 3). Before the official test, a preload of 10 N was applied three times at the same velocity (2 mm/min) to the radial head fragment. This position was regarded as the baseline to record the displacement of the fragment. The data was cleared from the strain analyzer. The construct was then loaded in compression at the rate of 2 mm/min. The test stopped when the load was 1 mm from the baseline.

After the axial load test, if the fracture models did not fail, they were performed to the failure load at the rate of 2 mm/min. In our text, all 24 fracture models did not fail. Failure was defined as a new fracture seen on the radial bone, an acute change in the load-displacement curve indicating a rapid change in displacement and loss of construct stability, or radial head displacement greater than 2 mm (the fracture model had an intra-articular fracture, and the failure of intra-articular fracture fixation is defined as a displacement of more than 2 mm). Axial stiffness and axial failure loads were recorded.

The stiffness was determined from the slope of the regression line fitted to the loading segment of the cyclic load-displacement curves. Data from each group are presented as the mean ± standard deviation. SPSS 21.0 (IBM Corporation, Armonk, NY, USA) was used for statistical analysis. Mechanical parameters were compared using an LDS-test. *p* < 0.05 was considered statistically significant.

## Results

**Fig. 4:**
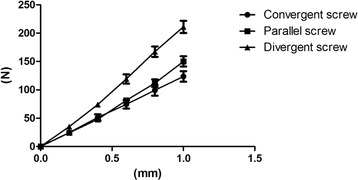
Comparison the axial stiffness of 24 specimens between convergent group, parallel group, and divergent group. The slopes of the curves reflect the stiffness of three groups

We analyzed the stiffness of the three groups from five levels. All the bending load data was collected and processed as the load-displacement curve seen in Fig. 4. The three curves in the figure represent three different load-displacement variations. It is easy to see that the load-displacement variations for the three groups were approximately linear in the range of 0–1 mm. The slope of the fold line represents the stiffness of the implant. As we can see from Table 1, the divergent group was the hardest with a stiffness of 213.9 ± 28.00 N/mm, and the stiffness of the convergent group was 123.5 ± 25.94 N/mm (*p* < 0.05). The stiffness of the parallel group was 149.5 ± 23.32 N/mm (*p* < 0.05). Although the stiffness of the parallel group was higher than that of the convergent group, there was no statistically significant difference between the two groups (*p* > 0.05).

**Table 1:** Average stiffness on axial load test of parallel group, convergent group, and divergent group

	Convergent group	Parallel group	Divergent group
Average stiffness(N/mm)	123.5 ± 25.94	149.5 ± 23.32	213.9 ± 28.00

**Fig. 5:**
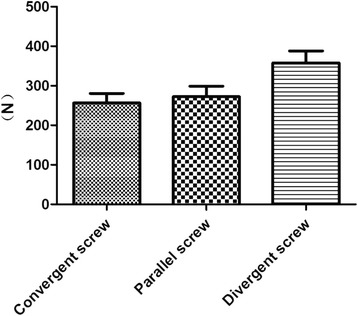
Comparison of axial failure load between convergent group (8 specimens), parallel group (8 specimens), and divergent group (8 specimens). Standard deviation is represented with the range bars on top of each graph

The three groups did not fail when loading to the displacement of 1 mm, so we selected the displacement of 2 mm as the failure load. All fracture models were not failure after the axial load test and 24 specimens were subjected to failure load tests. The divergent screws were still the hardest implant with a failure load of 357.6 ± 74.58 N, and they had statistically significant differences from the other two groups. The load of the convergent screws was 256.5 ± 59.53 N, which was 39.44% smaller than that of the divergent screws, and the load of the parallel screws was 272.3 ± 65.46 N, which was 31.33% smaller than that of the divergent screws (Fig. 5).

## Discussion

The results obtained via conservative treatment may be satisfactory if the fracture is not displaced or is minimally displaced but movement is not impeded [1]. In recent years, it has been generally acknowledged that these fractures are best treated by open reduction with internal fixation if the fragment is displaced more than 2 mm or involves more than 30% of the radial head. In a study by Demiroglu et al. [13], 23 patients were treated operatively with screw fixations with a follow-up period of more than 11 months. Their study showed that anatomical reduction of type II radial head fractures through open surgery and fixed with screws can have favorable results. Similarly, Pearce et al. [14] used Herbert screws to treat radial head fractures with satisfactory results. Burkhart et al. [15] recommended surgery to avoid the development of post-traumatic arthritis. Van Glabbeek et al. [16] also reported satisfactory results with open reduction of Mason type II radial head fractures. Consistent with these findings, the common interest in preservation of the radial head is steadily increasing. New techniques and implants have been developed for the maintenance of radial head fractures. However, only a few biomechanical studies have examined Mason type II radial head fractures to date [17–22]. Klaus et al. [17] evaluated the 3.0-mm headless compression screw and the standard 2.0-mm cortical screw used for fixation of radial head fractures. No significant differences concerning the stability achieved by the 3.0-mm headless compression screw, and the 2.0-mm cortical screw could be detected in the experimental setup presented. In a recent article, Christina et al. [22] compared the mechanical properties of crossed screw and plate fixation in the model of a radial neck fracture; the two strategies provided similar strength and stiffness for the fixation of transverse, non-comminuted radial neck fractures.

In a recent article, Jeffrey et al. [23] evaluated high-fidelity composite bone models used in the biomechanics study; Synbone brand models was well represented in the hand and upper extremity biomechanics research. In our biomechanical experiments, the divergent screw group showed the highest axial compression stiffness of the three groups; in contrast, the axial compression stiffness of the convergent group was the lowest of the three groups, which was consistent with our expected results. Amanatullah et al. [24] evaluated the mechanical properties of three different screw orientations used for the fixation of vertical shear fractures of the medial malleolus. The use of a divergent screw pattern resulted in a stiffer fixation construct when used to stabilize an osteotomy model of vertical shear medial malleolus fractures. In their study, it was noted that when the screws were not placed in parallel only the first screw caused compression, and any non-parallel screws did not add additional compression, but instead acted as a rotation, translation, and tensile force neutralizer. However, in a non-rigid system, each screw provides additional compression and acts as a rotation, translation, and tensile force neutralizer. Similar results can be drawn from our study and their study: divergent screw placement allows the screws to be farther apart in the fracture plane. This wider screw orientation encompasses a larger surface of bone in the fracture plane that is held in compression and resists translation in the axial plane and rotation in the sagittal plane as a result of increased interfragmentary friction.

According to our biomechanical results, the divergent screws showed the greatest axial stiffness in our standard Mason type II radial head fracture model. It is envisaged that in clinical applications, no matter what the direction of the two screws, the soft tissue detachment is similar and only a small range of stripping is needed. Therefore, if divergent screws are applied in the clinical setting, their effect may be better than the clinical use of parallel screws. Early postoperative exercise after radial head fractures is the basic factor of postoperative rehabilitation [13]. Stronger stiffness can be better for fixing the fracture block and preventing displacement of the fracture block after surgery, to achieve earlier and better postoperative rehabilitation exercise and prevent postoperative complications. However, this is only our vision; the feasibility requires a large amount of clinical validation. We can only form a purely biomechanical point of view, and pathogenesis and other internal fixation mechanisms need to be taken into consideration for the treatment of radial head fractures.

Our study also had some limitations. First, the standard bone without muscle and other corresponding soft tissue attachment cannot simulate the force transmission and role of the real human elbow joint. Second, our sample size in the study was not big enough; axial load direction cannot completely simulate the real daily activities of the human body or the mechanical mechanism of the injury. What is more, the load application is not representative of how load is transferred through the elbow. Finally, the biomechanics of this study only included axial and failure loads; the observed index was only axial stiffness due to the lack of more biomechanical performance indicators.

## Conclusion

Our findings demonstrated that the divergent screws had more biomechanical advantages over the other two screw orientations. However, our conclusion needs to be supported by additional studies with large sample sizes looking at biomechanical and clinical applications.

## References

[1] Mason ML (1954). Some observations on fractures of the head of the radius with a review of one hundred cases. Br J Surg.

[2] Broberg MA, Morrey BF (1987). Results of treatment of fracture-dislocations of the elbow. Clin Orthop Relat Res.

[3] Shulman BS, Lee JH, Liporace FA, Egol KA (2015). Minimally displaced radial head/neck fractures (Mason type-I, OTA types 21A2.2 and 21B2.1): are we “over treating” our patients?. J Orthop Trauma.

[4] Yoon A, Athwal GS, Faber KJ, King GJ (2012). Radial head fractures. J Hand Surg Am..

[5] Duckworth AD, Clement ND, Jenkins P, Aitken SA, Court-Brown CM, McQueen MM (2012). The epidemiology of radial head and neck fractures. J Hand Surg Am..

[6] Johnston GW (1962). A follow-up of one hundred cases of fracture of the head of the radius with a review of the literature. Ulster Med J.

[7] Esser RD, Davis S, Taavao T (1995). Fractures of the radial head treated by internal fixation: late results in 26 cases. J Orthop Trauma.

[8] Nalbantoglu U, Kocaoglu B, Gereli A, Aktas S, Guven O (2007). Open reduction and internal fixation of Mason type III radial head fractures with and without an associated elbow dislocation. J Hand Surg Am..

[9] Cobb TK, Beckenbaugh RD (1998). Nonunion of the radial neck following fracture of the radial head and neck: case reports and a review of the literature. Orthopedics.

[10] Faber FW, Verhaar JA (1995). Nonunion of radial neck fracture. An unusual differential diagnosis of tennis elbow, a case report. Acta Orthop Scand.

[11] Faraj AA, Livesly P, Branfoot T (1999). Nonunion of fracture of the neck of the radius: a report of three cases. J Orthop Trauma.

[12] Ring D, Quintero J, Jupiter JB (2002). Open reduction and internal fixation of fractures of the radial head. J Bone Joint Surg Am.

[13] Demiroglu M, Ozturk K, Baydar M, Kumbuloglu OF, Sencan A, Aykut S, Kilic B (2016). Results of screw fixation in Mason type II radial head fractures. Spring.

[14] Pearce MS, Gallannaugh SC (1996). Mason type II radial head fractures fixed with Herbert bone screws. J R Soc Med.

[15] Burkhart KJ, Wegmann K, Muller LP, Gohlke FE (2015). Fractures of the radial head. Hand Clin.

[16] Van Glabbeek F, Van Riet R, Verstreken J (2001). Current concepts in the treatment of radial head fractures in the adult. A clinical and biomechanical approach. Acta Orthop Belg.

[17] Burkhart KJ, Nowak TE, Appelmann P, Sternstein W, Rommens PM, Mueller LP (2010). Screw fixation of radial head fractures: compression screw versus lag screw—a biomechanical comparison. Injury.

[18] Burkhart KJ, Mueller LP, Krezdorn D, Appelmann P, Prommersberger KJ, Sternstein W, Rommens PM (2007). Stability of radial head and neck fractures: a biomechanical study of six fixation constructs with consideration of three locking plates. J Hand Surg Am..

[19] Capo JT, Svach D, Ahsgar J, Orillaza NS, Sabatino CT. Biomechanical stability of different fixation constructs for ORIF of radial neck fractures. Orthopedics. 2008;31(10). 19226014

[20] Giffin JR, King GJ, Patterson SD, Johnson JA (2004). Internal fixation of radial neck fractures: an in vitro biomechanical analysis. Clin Biomech (Bristol, Avon).

[21] Patterson JD, Jones CK, Glisson RR, Caputo AE, Goetz TJ, Goldner RD (2001). Stiffness of simulated radial neck fractures fixed with 4 different devices. J Shoulder Elb Surg.

[22] Gutowski CJ, Darvish K, Ilyas AM, Jones CM (2015). Comparison of crossed screw versus plate fixation for radial neck fractures. Clin Biomech (Bristol, Avon).

[23] Reed JD, Stanbury SJ, Menorca RM, Elfar JC (2013). The emerging utility of composite bone models in biomechanical studies of the hand and upper extremity. J Hand Surg Am.

[24] Amanatullah DF, Khan SN, Curtiss S, Wolinsky PR (2012). Effect of divergent screw fixation in vertical medial malleolus fractures. J Trauma Acute Care Surg.

